# A nonsense mutation in *PLD4* is associated with a zinc deficiency-like syndrome in Fleckvieh cattle

**DOI:** 10.1186/1471-2164-15-623

**Published:** 2014-07-22

**Authors:** Simone Jung, Hubert Pausch, Martin C Langenmayer, Hermann Schwarzenbacher, Monir Majzoub-Altweck, Nicole S Gollnick, Ruedi Fries

**Affiliations:** Chair of Animal Breeding, Technische Universitaet Muenchen, 85354 Freising, Germany; Clinic for Ruminants with Ambulatory and Herd Health Services at the Centre for Clinical Veterinary Medicine, Ludwig-Maximilians-Universitaet Muenchen, 85764 Oberschleissheim, Germany; Institute of Veterinary Pathology at the Centre for Clinical Veterinary Medicine, Ludwig- Maximilians-Universitaet Muenchen, 80539 Munich, Germany; ZuchtData EDV-Dienstleistungen GmbH, 1200 Wien, Austria

**Keywords:** Autozygosity mapping, Next-generation sequencing, Recessive inheritance, Zinc deficiency-like, Fleckvieh, Nonsense mutation, *PLD4*

## Abstract

**Background:**

Bovine hereditary zinc deficiency (BHZD) is an autosomal recessive disorder of cattle, first described in Holstein-Friesian animals. Affected calves suffer from severe skin lesions and show a poor general health status. Recently, eight calves with the phenotypic appearance of BHZD have been reported in the Fleckvieh cattle population.

**Results:**

In spite of the similar disease phenotypes, *SLC39A4,* the gene responsible for BHZD in Holstein-Friesian was excluded as underlying gene for the disorder in the affected Fleckvieh calves. In order to identify the disease-associated region, genotypes of eight affected calves obtained with the Illumina BovineHD BeadChip comprising 777,962 SNPs were contrasted with the genotypes of 1,339 unaffected animals. A strong association signal was observed on chromosome 21 (P = 5.87 × 10^-89^). Autozygosity mapping in the eight affected animals revealed a common segment of extended homozygosity encompassing 1,023 kb (BTA 21: 70,550,045 - 71,573,501). This region contains 17 genes/transcripts, among them two genes encoding gastro-intestinal zinc transporters (*CRIP1*, *CRIP2*). However, no mutation that was compatible with recessive inheritance could be detected in these candidate genes. One of the affected calves was re-sequenced together with 42 unaffected Fleckvieh animals. Analysis of the sequencing data revealed a nonsense mutation (p.W215X) in a phospholipase encoding gene (*PLD4*) as candidate causal polymorphism. To confirm the causality, genotypes of the p.W215X-mutation were obtained from 3,650 animals representing three different breeds. None of the unaffected animals was homozygous for the defect allele, while all eight affected calves were homozygous. The deleterious effect of the mutation is manifested in a significantly lower survival rate of descendants from risk matings when compared with the survival rate of descendants from non-risk matings. The deleterious allele has an estimated frequency of 1.1% in the Fleckvieh population.

**Conclusion:**

Our results provide strong evidence that a newly identified recessive disorder in the Fleckvieh population is caused by a nonsense mutation in *PLD4*, most likely resulting in an impaired function of the encoded protein. Although the phenotype of affected calves strongly resembles BHZD, a zinc deficiency resulting from malabsorption is unlikely to be responsible for the diseased Fleckvieh calves.

**Electronic supplementary material:**

The online version of this article (doi:10.1186/1471-2164-15-623) contains supplementary material, which is available to authorized users.

## Background

So far, 415 congenital defects have been identified in cattle, 109 of them being inherited in an autosomal recessive mode [1]. The widespread use of elite sires by means of artificial insemination makes cattle populations highly susceptible to the propagation of recessively inherited disorders [2]. However, the availability of dense SNP genotyping arrays facilitates the rapid identification of underlying genomic regions [3].

This paper details a disorder that resembles Bovine Hereditary Zinc Deficiency (BHZD, OMIA 000593-9913), an autosomal recessive condition, primarily seen in Holstein-Friesian calves [4]. This disease is characterized by an impaired function of the immune system, growth retardation and skin alterations as a result of a deficient gastrointestinal zinc absorption [5]. While affected animals are born without apparent clinical symptoms, first skin lesions emerge between the age of four and eight weeks [4]. Impairment of immune functions makes affected animals more susceptible to infectious pathogens and leads to an increased incidence of common calf diseases, *e.g.,* enteritis and pneumonia [6]. Highly-dosed oral zinc supplementation ameliorates clinical symptoms in affected Holstein-Friesian animals, however, if untreated, BHZD is lethal [5]. Inherited zinc absorption disorders, caused by mutations in the zinc transporter encoding gene *SLC39A4*, have been reported for several mammalian species, including mouse and human [7–9]. In Holstein-Friesian cattle, BHZD is caused by a splice-site variant in *SLC39A4* [8]. Hereditary zinc absorption disorders have not yet been described in the Fleckvieh population.

Here we show that a phenotype of Fleckvieh calves that resembles BHZD is not caused by a defective zinc transporter gene. Genome-wide association analysis, autozygosity mapping and analysis of whole-genome sequencing data enabled to identify a nonsense mutation in the phospholipase D family member 4 encoding gene (*PLD4*) which is likely causal for the observed phenotype.

## Results

### Phenotypic manifestation of the defect

**Figure 1:**
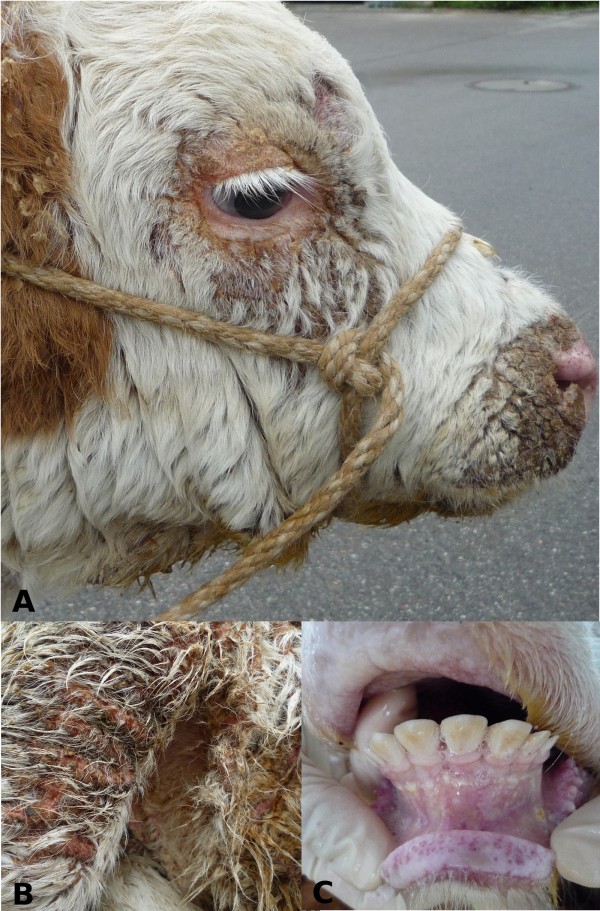
**Phenotypic manifestation of the newly identified disease in Fleckvieh calves.** A two month old male Fleckvieh calf with distinct scaling and crusting of the skin around the muzzle, eyes **(A)** and in the femoral region of the right hind leg **(B)**. Alterations due to seborrheic dermatitis can be noted in the inguinal region **(B)**. Erosive lesions of the oral mucosa in a 4.4-month old female patient **(C)**.

Seven calves between the age of seven and 17 weeks with severe skin lesions and poor general health status were admitted to the Clinic of Ruminants. Clinical findings such as scaling and crusting of skin and secondary adhesion of hair were most evident around the muzzle, the eyes, above the sternum and on the extremities (Figure 1A,B). Skin of the inguinal region was seborrhoeic and all palpable lymph nodes were enlarged. The calves suffered from erosions in the interdigital spaces and erosive or ulcerative lesions of the oral mucosa (Figure 1C). All animals were underdeveloped in height and weight and had a history of recurring diarrhoea and pneumonia. Two of the calves were supplemented with dietary zinc (750 mg/day), but did not respond to the treatment. Due to the advanced state of the disease and with no prospect for improvement, all animals were euthanized and subjected to necropsy.

**Figure 2:**
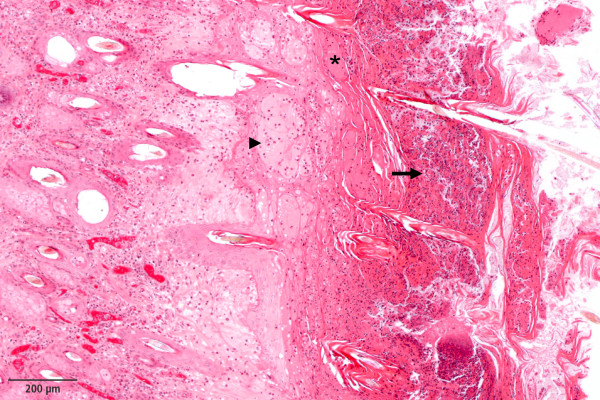
**Hematoxylin and eosin (H&E) stained dermal section of an affected calf.** Severe epidermal vesiculation (arrowhead), multiple intracorneal serum accumulations (asterisk), serocellular crusts (arrow) and diffuse dermal oedema and inflammation, plastic embedding technique, H&E stain.

In hematoxylin and eosin (H&E) stained sections, the skin of the affected animals displayed a mild to severe chronic dermatitis with serocellular crusting and partial superficial bacterial colonization. Multiple intracorneal and intraepidermal accumulations of serum as well as multiple ulcerations and epidermal necroses were present. The epidermis was severely oedematous in multiple sections (Figure 2). Dermis and epidermis were diffusely infiltrated by a moderate to high number of neutrophils. Besides moderate oedema, the dermis exhibited infiltration with lymphocytes and plasma cells to a limited extent. In the cases where the thymus was examined, decreased thymic cellularity and poorly demarcated cortex-medulla-border were observed. Further, the clinical diagnoses of enteritis and pneumonia were confirmed during the pathological examination.

In addition to the seven cases described above, another Fleckvieh calf (eleven weeks old) with severe alterations of the skin and diarrhoea was reported by a veterinarian. The described lesions were very similar to the phenotype of the previously reported seven calves. The calf received dietary zinc (750 mg/day) for a period of two weeks. However, the overall condition of the animal did not improve and the calf died at the age of 13 weeks.

Inspection of the pedigrees of the affected calves revealed a common ancestor suggesting a genetic background. On the basis of the patients’ history, the clinical and pathological findings, the calves were tentatively diagnosed to suffer from BHZD.

### Analysis of *SLC39A4 –*the gene causing BHZD in Holstein-Friesian

Mutations in *SLC39A4* are known to cause defects resembling the phenotypic appearance of the eight affected Fleckvieh calves in various species including cattle [8]. *SLC39A4* is located at the proximal region of bovine chromosome 14 (BTA 14: 1,719,732 bp – 1,724,221 bp). The gene was re-sequenced in a case–control panel consisting of all affected animals, all available dams and sires and randomly selected, unaffected control animals. Totally ~7 kb of genomic sequence was screened, resulting in the detection of ten SNPs (Additional file 1). The mutation causing BHZD in Holstein-Friesian was not present in the diseased animals and none of the detected polymorphisms was associated with the disease phenotype, nor was any of the polymorphic sites compatible with the supposed pattern of recessive inheritance.

### Identification of the disease-associated region

**Figure 3:**
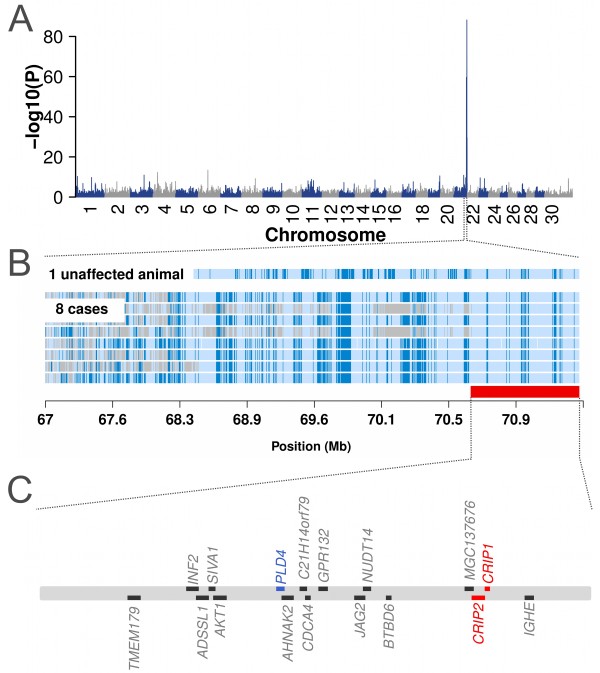
**Mapping of the locus for a zinc deficiency-like syndrome in the Fleckvieh population.** Association of 644,450 SNPs with the affection status of eight affected and 1,339 unaffected Fleckvieh animals **(A)**. P values were obtained by fitting a linear mixed model. Autozygosity mapping in eight affected calves and one unaffected animal **(B)**. Blue and pale blue represent homozygous genotypes (AA and BB), heterozygous genotypes (AB) are displayed in light grey. The red bar indicates the common segment of homozygosity. Note that one unaffected animal is also homozygous for the segment of extended homozygosity. The segment of extended homozygosity encompasses 17 transcripts/genes among them *CRIP1, CRIP2* and *PLD4* **(C)**.

Since the analysis of *SLC39A4* did not reveal a potentially causal mutation, we applied an array-based approach to identify the underlying genomic region. The eight affected calves together with 1,339 unaffected Fleckvieh bulls were genotyped with the Illumina BovineHD BeadChip. A genome-wide association study using genotypes of 644,450 SNPs revealed a strong association signal on BTA 21. Eighty-two SNPs located within an 18.19 Mb interval from 53,140,245 bp to 71,333,740 bp were significantly associated (P < 7.88 × 10^-8^) (Figure 3A). The most prominent association signal (P = 5.87 × 10^-89^) was observed for *BovineHD4100015383*, located at 69,873,257 bp. As expected, the proximal region of BTA 14 harbouring *SLC39A4* did not show association at all.

Autozygosity mapping within the distal region of BTA 21 revealed a common 1,023 kb segment of extended homozygosity in the eight affected animals (70,550,045 bp – 71,573,501 bp) (Figure 3B). However, one out of 1,339 animals of the control group was homozygous for the disease-associated region as well. Signal intensities obtained from high-density genotyping revealed no indication for the presence of large structural variants (deletions, copy number variations) within the associated region (Additional file 2).

The frequency of the associated haplotype was estimated in a sample of 10,355 unaffected Fleckvieh animals. Among them, 380 were heterozygous carriers and three were homozygous, yielding a frequency for the associated haplotype of 1.86%. The haplotype distribution shows no deviation from the Hardy-Weinberg equilibrium (P = 0.75).

### Analysis of *CRIP1*and *CRIP2*– genes with similar function as *SLC39A4*

The segment of extended homozygosity contains 17 transcripts and protein encoding genes, respectively (UMD3.1 annotation [10], Figure 3C). Two of these genes encode cysteine-rich proteins (*CRIP1* and *CRIP2*). *CRIPs* are highly expressed in the intestine [11] and are essential for zinc absorption as well as for immune system related cytokine regulation [12, 13]. Thus, they represent excellent candidate genes for the observed disease phenotype.

*CRIP1* and *CRIP2* (71,390,596 bp – 71,392,110 bp and 71,365,858 bp – 71,382,666 bp, respectively) were re-sequenced in the case–control panel. We screened ~6 kb of the genomic sequence of *CRIP1*, resulting in the detection of 18 SNPs and two insertion/deletion (InDel) polymorphisms (Additional file 1). Approximately 8 kb of the genomic sequence of *CRIP2* were screened, resulting in the identification of 30 SNPs (Additional file 1). None of the variants in *CRIP1* and *CRIP2* was compatible with the disease phenotype. Thus, variation in the two positional and functional candidate genes is likely not causal for the observed disease.

### Identification of the underlying mutation by exploiting whole-genome sequencing data

In a next attempt to identify the causal mutation, one of the affected calves (id = 58953) and one of the unaffected homozygous animals (id = 58952) were re-sequenced together with 41 animals of the FV population [14]. Multi-sample variant calling yielded genotypes for 7,660 polymorphic sites within the 1,032 kb disease-associated segment at the distal end of BTA 21. Sequence coverage in the disease-associated region averaged 5.19x and 3.95x for 58953 and 58952, respectively with 87.95% and 80.60% of the positions being covered with more than one read. Haplotype analysis revealed that none of 41 re-sequenced control animals carried the disease-associated haplotype. Three SNPs and one InDel were compatible with recessive inheritance, *i.e.*, they were homozygous for the alternative allele (aa) in the affected calf, heterozygous (Aa) or homozygous for the reference allele (AA) in 58952 and homozygous for the reference allele (AA) in all 41 remaining animals (Table 1). None of the four polymorphisms were located within the coding regions of two candidate genes *CRIP1* and *CRIP2*.

**Table 1:** **Genotypes of four variants compatible with recessive inheritance**

Chr	Position	NCBI reference ID	Variant	Genotypes			Gene	Effect
				Calf	58952	41 animals		
21	70,679,787	*rs381259516*	InDel	del/del	del/G	G/G	intergenic	-
21	70,842,696	*rs384306864*	SNP	TT	CC	CC	*INF2*	p.H1231Y
21	71,001,232	*rs378824791*	SNP	AA	GG	GG	*PLD4*	p.W215X
21	71,315,111	*rs385301007*	SNP	TT	CC	CC	*LOC100299595 (=PACS2)*	intron 30

Whole-genome re-sequencing of 43 animals revealed 7,660 polymorphic sites within the 1,032 kb segment on BTA 21. Among them, three SNPs and one InDel agree with the supposed inheritance pattern (*i.e.,* the affected calf is homozygous for the alternative allele and the controls are heterozygous or homozygous for the reference allele). 58952 is a healthy animal being homozygous for the disease-associated haplotype.

**Figure 4:**
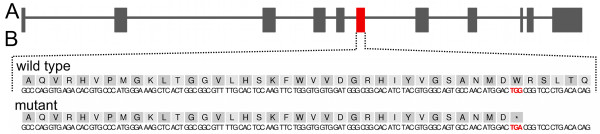
**A nonsense mutation in** ***PLD4*** **is perfectly associated with the disease phenotype.** Genomic structure of bovine *PLD4* **(A)**. Grey boxes represent exons. The red box represents exon 6 including *rs378824791*, introducing a premature stop codon. Genomic and protein sequence of exon 6 of *PLD4* **(B)**. The affected codon (p.W215X, TGG → TGA) is highlighted with red colour.

The compatible InDel-polymorphism (*rs381259516*) is also segregating among 191 non-Fleckvieh animals (128 Holstein, 15 Jersey, 48 Angus), which have been sequenced in the context of the 1000 bull genomes project [15]. No regulatory or functional consequence was predicted for the compatible SNP (*rs385301007*) in intron 30 of *LOC100299595*. Manual re-annotation of *INF2* revealed that the presumed missense mutation (*rs384306864*) is not located within the coding region of the gene (Additional file 3). Therefore, these three variants were excluded as being causal for the described phenotype. Only a point mutation in exon 6 of *PLD4,* resulting in a premature stop codon (c.G645A, p.W215X, BTA 21:71,001,232 bp, *rs378824791*) (Figure 4, Additional file 3), was retained as candidate causal mutation. The resulting protein is shortened by 273 amino acids and lacks essential domains for enzymatic activity [16, 17]. The mutation was confirmed by Sanger sequencing (Additional file 4).

### Validation of the p.W215X-mutation in *PLD4*

Genotypes of the p.W215X-mutation (*rs378824791*) were obtained for 3,650 animals representing three different breeds (Fleckvieh: 3,088, Braunvieh: 280, Holstein-Friesian: 282). All eight affected calves were homozygous for the mutation, while none of the unaffected animals was homozygous (Table 2). The mutation does not segregate in Braunvieh and Holstein-Friesian. Three unaffected animals identified as homozygous for the disease-associated haplotype *via* array-derived genotypes were heterozygous and homozygous for the reference allele, respectively. We obtained genotypes for 169 animals that are heterozygous carriers of the disease-associated haplotype (identified *via* array-derived genotypes). Among them, only 100 carry the mutation while 69 are homozygous for the reference allele. The frequency of the associated haplotype was estimated to be 1.86% based on array-derived genotypes (see above). However, only 59.5% of the animals carrying the disease haplotype also carry the p.W215X-mutation. Thus, the frequency of the defect allele amounts to 1.1% in the Fleckvieh population.

**Table 2:** **Genotypes for the p.W215X-mutation in** ***PLD4*** **for 3,650 animals**

Breed	Animal group	Samples	Genotypes for p.W215X		
			AA	AG	GG
FV	Affected calves	8	8	-	-
	Dams of the calves	6	-	6	-
	Unaffected animals being homozygous for the disease-associated haplotype	3	-	2	1
	Animals carrying the disease-associated haplotype	169	-	100	69
	Animals not carrying the disease-associated haplotype	1,777	-	-	1,777
	Animals with unknown haplotype status	1,125	-	51	1,074
HF	Randomly selected animals	282	-	-	282
BV	Randomly selected animals	280	-	-	280

Genotypes for the nonsense-mutation (*rs378824791*) were obtained for 3,650 animals representing three different breeds (Fleckvieh (FV), Holstein-Friesian (HF) and Braunvieh (BV)) using a TaqMan^®^ genotyping assay. The nonsense-mutation is homozygous in the affected calves only. The mutation does not segregate in Holstein-Friesian and Braunvieh. Note that animals carrying the disease haplotype were specifically added to Fleckvieh panel.

### Survival rate of progeny descending from bulls carrying the p.W215X-mutation

**Figure 5:**
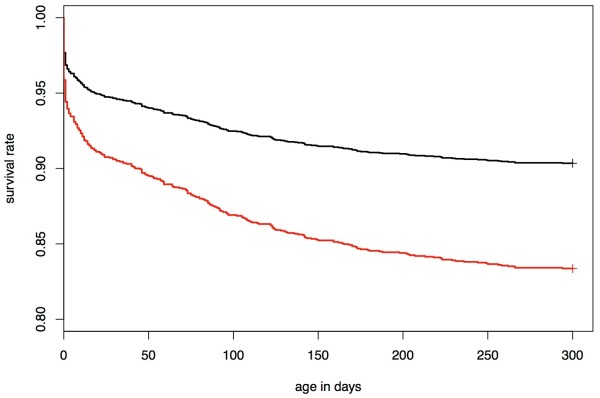
**Survival analysis of calves from different mating types.** Survival rate of calves from two different mating types (non-carrier [sire] × carrier [maternal grandsire], carrier [sire] × carrier [maternal grandsire]) as a function of the animals' age. The black line represents the survival rate of calves descending from matings where the maternal grandsire is a carrier while the sire does not carry the p.W215X-mutation. The red line represents the survival rate of calves descending from matings where both, sire and maternal grandsire are carriers of the mutation.

In order to study the effect of the deleterious allele on the population level, we estimated the survival rate of the descendants from different mating types with regard to the p.W215X-genotype in sires and maternal grandsires. If both, sire and maternal grandsire are heterozygous for the p.W215X-mutation, the probability of the resulting calf being homozygous is 12.5%. The survival rate of calves descending from such matings (N = 1,213) is significantly lower (P = 2.971 × 10^-8^) compared to the survival rate of calves descending from non-risk matings across all age classes, as expected in the case of recessive inheritance of the deleterious allele (Figure 5). At day 300, 16.63% of the calves resulting from risk matings have perished, while the mortality of calves descending from non-risk matings is 9.66% only (Table 3).

**Table 3:** **Survival rate of calves descending from different mating types**

Carrier-state for the p.W215X-mutation		Number of calves	Survival rate at different days						
Sire	Maternal grandsire		6	10	20	50	102	202	300
Non-carrier	Carrier	2,552	0.961	0.957	0.949	0.940	0.924	0.909	0.903
Carrier	Carrier	1,213	0.931	0.923	0.911	0.895	0.869	0.843	0.834

The survival rate of calves from different mating types was estimated using a Kaplan-Meier estimator.

## Discussion

Eight calves with severe skin lesions and poor general health status were recently identified in the Fleckvieh cattle population. Clinical and pathological examinations of affected animals showed striking similarities to findings described for individuals suffering from zinc deficiency in cattle [4, 6, 18] and humans [19]. Inherited zinc absorption disorders, resulting from mutations in *SLC39A4* [8], can be ameliorated by highly-dosed oral zinc supplementation [5]. However the Fleckvieh calves did not respond to oral zinc supplementation. Variants in *SLC39A4* could be excluded from being causal for the phenotype of the affected Fleckvieh animals, suggesting a different aetiological basis of the syndrome observed in the Fleckvieh calves.

Genome-wide association analysis followed by autozygosity mapping identified a common 1,023 kb segment of extended homozygosity on BTA 21 in the affected calves. Variants in two genes (*CRIP1* and *CRIP2*) with functional similarity to *SLC39A4* [20, 21] were excluded to be associated with the phenotype of the affected calves. Finally, genome-wide re-sequencing of one of the affected calves revealed a putatively causal loss-of-function mutation in the phospholipase D family member 4 encoding gene *PLD4*. Given the relatively low fold sequence coverage, some variants might have been wrongly called and lost, respectively. However, multi-sample variant calling followed by population-based genotype imputation as applied in the present study provides high quality genotypes even for lowly covered sites [14]. The p.W125X-mutation was perfectly associated in a panel of 3,650 animals, further substantiating our suspicion of causality.

Phospholipase D4 is a member of the family of phospholipid signalling enzymes [17] and is mainly expressed in spleen and early microglia, suggesting a role in immunological pathways [22, 23]. Although PLD4 seems to be involved in phagocytosis, little is known about its enzymatic function in cells [22]. Recent studies revealed an association between variants in *PLD4* and two autoimmune diseases in human, namely systemic sclerosis [23] and rheumatoid arthritis [24], disorders with inflammatory skin lesions [25] and interstitial lung diseases [26, 27]. Furthermore, knock-out mice with a nonsense mutation in *PLD4* manifest a phenotype with thin hair and growth retardation [28]. In addition, Arun *et al.* [29] demonstrated the importance of phospholipase family D members in mediating the repair of plasma membrane disruptions in mice keratinocytes emphasising the role of these phospholipid signalling enzymes in membrane function and wound healing. A mutation in a phospholipase-domain containing protein (*PNPLA1*) results in a severe cornification disorder in dogs, providing evidence for a key role of lipases in the keratinisation process and the metabolism of the epidermal barrier in general [30]. Taken together, it seems very likely that an impaired function of PLD4 is causal for the severe skin lesions and the poor general health status of the affected calves.

Compared to the wild-type protein, the PLD4 protein is predicted to be shortened by 273 amino acids in the affected calves. The truncated protein may be retained with an impaired function and/or the transcript may be degraded *via* nonsense-mediated mRNA decay [31]. If the truncated protein is retained, its function is likely to be severely compromised as it lacks essential domains for enzymatic activity [16, 17]. However, the actual effect of the mutation on the expression of *PLD4* needs to be unravelled in subsequent studies.

Although there is a striking similarity in the phenotypic appearance of the Fleckvieh calves and Holstein-Friesian animals suffering from BHZD [4], there are no clues for a connection between PLD4 and zinc metabolism. Furthermore, zinc supplementation did not ameliorate any of the symptoms of the affected calves, corroborating that impaired zinc metabolism might not be causal for the phenotype. Based on our findings the tentative diagnosis of zinc deficiency can no longer be maintained.

Haplotype analysis revealed that three unaffected animals are homozygous for the associated haplotype. However, none of them was homozygous for the p.W215X-mutation. It seems likely that the mutation might have occurred in the germline of a recent founder animal, resulting in two identical haplotypes differing for the deleterious allele only. A similar situation has been observed for the arachnomelia syndrome (OMIA 000059-9913) in Brown Swiss cattle [32]. Using array-derived genotypes does not allow distinguishing between animals carrying the affected and unaffected haplotype version. Thus, the haplotype frequency derived from array-based genotypes (1.86%) is higher than the frequency of the p.W125X-mutation. The frequency of the p.W215X-mutation was estimated to 1.1% only in the current Fleckvieh population. However, the widespread use of a single bull carrying the defect allele could lead to a rapid increase of the frequency within few generations. Since array-based genotypes are routinely obtained for all candidate bulls, animals carrying the disease-associated haplotype can now be easily identified. Such animals should be directly genotyped for the p.W215X-mutation and animals carrying the mutation should be excluded from artificial insemination. This cost-effective approach will prevent unneeded animal suffering and economic losses by avoiding inadvertent carrier x carrier matings.

Assuming a frequency of 1.1% of the deleterious allele in the Fleckvieh population, equal use of all bulls and ~1,000,000 annual births, one would expect 121 affected calves per year. The actual number of expected cases is probably somewhat lower, as most carriers are related and farmers avoid close inbreeding. Furthermore, most of these cases will probably not be reported since the affected calves die due to rather unspecific diseases (*e.g.,* enteritis or diarrhoea) before the manifestation of characteristic symptoms (*i.e.,* hyperkeratotic and fissured skin). The significantly reduced survival rate of descendants from risk matings supports this assumption. However, incomplete penetrance could also reduce the observed incidences. Therefore it will be important to study animals being homozygous for the identified mutation from birth in a controlled environment for a better characterisation of the disorder.

## Conclusion

A recessively inherited condition in Fleckvieh cattle resembles the phenotypic appearance of bovine hereditary zinc deficiency. Our results strongly support that a nonsense mutation (p.W215X) in the PLD4 encoding gene is causative for this disease in the Fleckvieh breed and that zinc deficiency is not involved in the aetiology of the disease. Further studies are necessary to unravel the detailed genotype-phenotype relationship. However, the identification of the causal variant allows for broad testing in the Fleckvieh population. Hence, an efficient management of this new defect is now possible.

## Methods

### Animal ethics statement

Semen samples were collected by approved commercial artificial insemination stations as part of their regular breeding and reproduction measures in cattle industry. The collection of blood samples was carried out by trained veterinarians during treatment of affected animals following standard veterinary protocols in Germany. No ethical approval was required for this study.

### Animals and DNA extraction

In order to confirm a genetic predisposition to the observed phenotype, a case–control panel consisting of the eight affected calves, their dams and sires and twelve unaffected control animals was set up. Seven affected animals were identified and examined by veterinarians of the Clinic for Ruminants. One affected calf was reported by a herd veterinarian. Blood samples of the affected calves and their dams were collected by trained veterinarians following standard procedures and DNA was extracted using proteinase K digestion and salt-out extraction. For the control group, genomic DNA was prepared from semen straws following standard protocols using proteinase K digestion and phenol-chloroform extraction.

### Annotation and polymorphism screening of *SLC39A4, CRIP1*and *CRIP2*

The *GENOMETHREADER* software tool [33] was used to predict the genomic structure and localization of *SLC39A4, CRIP1* and *CRIP2* based on the University of Maryland UMD3.1 assembly of the bovine genome sequence [10] and the Dana–Farber Cancer Institute bovine gene index release 12.0 [34] together with the annotated RNA sequences of the *UMD3.1* assembly [10]. The *GENOMETHREADER* output was viewed and edited using the *Apollo* sequence annotation editor [35]. The genes were PCR amplified (the primers are listed in Additional file 5), including exons, introns and the flanking 3'- and 5'-regions. Sequencing reactions were done using BigDye^®^ Terminator v1.1 Cycle Sequencing Kit (Applied Biosystems, Foster, CA., USA; Life Technologies Corporation, USA). Electrophoresis of purified sequencing reactions was performed on the ABI 3130x1 Genetic Analyzer (Applied Biosystems, Foster, CA., USA; Life Technologies Corporation, USA). The *Phred/Phrap/Polyphred* software suite [36–38] was used for base calling, sequence alignment and polymorphism detection. Sequences were viewed with *consed* [39].

### High- density genotyping and quality control

The eight affected calves and 1,339 unaffected Fleckvieh bulls were genotyped with the Illumina BovineHD BeadChip comprising 777,962 SNPs. Genotype calling was performed using default parameters of Illumina's *BeadStudio*. The chromosomal position of the SNPs was determined based on the UMD3.1 assembly of the bovine genome [40]. 343 mitochondrial, 1,224 Y-chromosomal and 1,735 SNPs with unknown chromosome position were not considered for subsequent analyses. Quality control was carried out with *PLINK* v1.07 [41]. 19,734 SNPs for which genotyping failed in more than 5% of the individuals and 110,746 SNPs with minor allele frequency < 0.5% were omitted. Sporadically missing genotypes were imputed using *Beagle* genetic analysis software [42]. The final dataset comprised 1,347 animals (eight affected calves and 1,339 controls) and 644,450 SNPs.

### Genome-wide association study

To account for population stratification and the resulting inflation of false positive associations, a mixed model based association analysis was performed. We used *GEMMA* [43] to fit the linear mixed model y = μ + xb + Zu + e, where y denotes the affection status (coded as 1 and 2 for affected and unaffected animals, respectively), μ is the intercept, x is a vector of marker genotypes, b is the SNP effect, Z is an incidence matrix, u is a vector of random polygenic effects ~ N(0, G), where  is the additive genetic variance and G is the genomic relationship matrix (GRM) among the 1,347 animals built based upon 627,627 autosomal SNPs following VanRaden's approach [44] and e is a vector of error terms.

### Haplotype analysis

Haplotypes of 1,347 animals of the initial genome-wide scan were inferred using default parameters of *Beagle* genetic analysis software (see above). To assess the frequency of the associated haplotype in a larger sample of the Fleckvieh population, array-derived genotypes of another 9,016 unaffected animals were analysed. Of the 9,016 animals, 7,000 were genotyped with the BovineSNP50 BeadChip (50 K) and 2,016 were genotyped with the Illumina BovineHD BeadChip (777 K). High-density genotypes and haplotypes of the 50 K data set were inferred based on haplotypes of the 777 K data set using a combination of *Beagle* (see above) and *Minimac* [45], which yields high imputation accuracy in cattle [46].

### Exploiting whole-genome re-sequencing data for mutation screening

The genomes of 43 animals of the Fleckvieh population were sequenced to an average coverage of 7.46x, among them an affected calf and a healthy animal being homozygous for the disease-associated haplotype with coverages of 7.8x and 6.2x, respectively. Sequencing on Illumina GA IIX and Hiseq 2000 instruments was performed as detailed by Jansen *et al.* [14]. Paired-end reads were obtained and mapped to the bovine reference sequence (see above) using the Burrows-Wheeler Aligner (*BWA*) [47]. *PICARD* (http://picard.sourceforge.net) was used to mark PCR-duplicates. Subsequent multi-sample variant calling with *mpileup* [48] yielded genotypes at 17.17 million sites. *Beagle* phasing and imputation was applied to improve the primary genotype calls. A detailed overview of the entire variant calling pipeline and all obtained variants is presented in Jansen *et al.* [14]. Of 17.17 million sites, 7,086 SNPs and 574 InDels were located within the 1,023 kb segment (70,550,045 bp – 71,573,501 bp) of extended homozygosity on BTA 21. To account for inaccurately genotyped variants due to the low-coverage sequence data (*e.g.,* mis-calling of heterozygous genotypes for rare variants [49, 50]), we filtered for variants that were segregating (heterozygous or homozygous for the non-reference allele) in the affected animal, heterozygous or homozygous for the reference allele in the healthy animal being homozygous for the disease-associated haplotype and homozygous for the reference allele in 41 healthy animals.

### 5'-exonuclease diagnostic assay of the c.G645A-*PLD4*mutation

A 5'-exonuclease assay was developed to obtain genotypes for the nonsense mutation (c.G645A, p.W215X, Chr21:71,001,232 bp, *rs378824791)*, using 5'-GGG CGG CAC ATC TAC GT-3' and 5'-CCA GGG CGG ACG AAC TC-3' as PCR primers, and 5'-CAT GGA CTG **G**CG GTC C-3' (wild type G allele) and 5'-CAT GGA CTG **A**CG GTC C-3' (mutant A allele) as probes (TaqMan^®^, Life Technologies Corporation, USA). Reactions were carried out on an ABI7500 Real-Time PCR system (Life Technologies Corporation, USA) using standard procedures and analysed using the allelic discrimination endpoint analysis mode of the 7500 software package v2.0.5. Genotypes for the polymorphism were obtained in 3,650 animals representing three different breeds (Fleckvieh, Holstein-Friesian, Braunvieh).

### Survival analysis

The survival rate of calves from two different mating types was estimated using a Kaplan-Meier estimator as implemented in the R package ('survival') [51]. For the control group the survival rate of 2,552 calves descending from matings where the sire does not carry the p.W215X-mutation whereas the maternal grandsire is a carrier S5(non-risk matings) was estimated. The case group comprised 1,213 calves descending from matings where both sire and maternal grandsire carry the p.W215X-mutation (risk-matings).

## Availability of supporting data

All relevant SNPs (with accession numbers of dbSNP) supporting the results of this article are included within the article and its additional files.

## Electronic supplementary material

Additional file 1: **Overview of the identified SNPs in** ***SLC39A4, CRIP1*** **and** ***CRIP2.*** (XLS 36 KB)

Additional file 2: **CNV-analysis within the segment of extended homozygosity.** Signal intensities obtained from genotyping with the Illumina BovineHD BeadChip are displayed as log R ratios for cases and (randomly selected) controls within the segment of extended homozygosity. The log R ratio is displayed for 3-SNP-sliding windows. (PNG 80 KB)

Additional file 3: **Genomic structure of the re-annotated** ***PLD4*** **and** ***INF2*** **genes.** The genomic structure was predicted based on the University of Maryland UMD3.1 assembly of the bovine genome sequences [10] and the Dana-Farber Cancer Institute bovine gene index release 12.0 [33] by using GENOMETHREADER software tool. The GENOMETHREADER output was viewed and edited using Apollo sequence annotation editor [35]. (PDF 46 KB)

Additional file 4: **Visualisation of the p.W215X mutation in two apparently homozygous animals using the integrative genomics viewer (IGV,** http://www.broadinstitute.org/igv/ **).** Analysis of whole-genome re-sequencing data revealed a nonsense mutation in *PLD4* as most likely causal for the disease of the affected calves. Re-sequencing confirmed that the mutation is homozygous in the affected calf. (PNG 258 KB)

Additional file 5: **List of primer pairs for** ***SLC39A4*** **,** ***CRIP1*** **and** ***CRIP2.*** (XLS 41 KB)
