# Supplementary figures and images for: A nonsense mutation in PLD4 is associated with a zinc deficiency-like syndrome in Fleckvieh cattle
Source: BMC Genomics. 2014 Jul 22;15(1):623. doi: 10.1186/1471-2164-15-623 (PMC4117962; doi:10.1186/1471-2164-15-623)

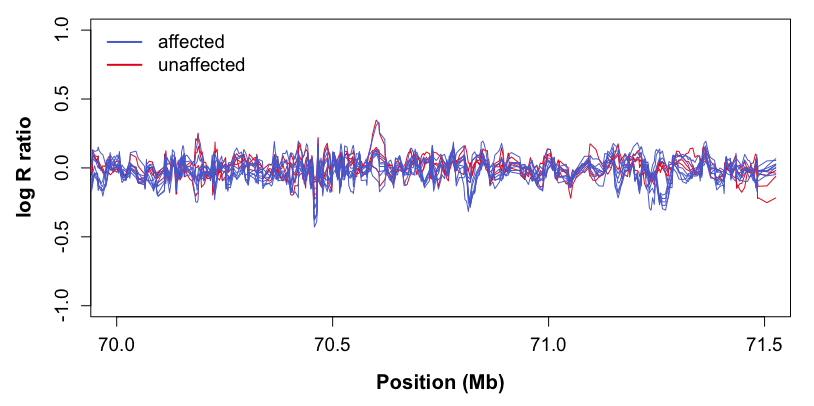

Supplement: Supplementary file 2 — Additional file 2: CNV-analysis within the segment of extended homozygosity. Signal intensities obtained from genotyping with the Illumina BovineHD BeadChip are displayed as log R ratios for cases and (randomly selected) controls within the segment of extended homozygosity. The log R ratio is displayed for 3-SNP-sliding windows. (PNG 80 KB) [file 12864_2014_6308_MOESM2_ESM.png]

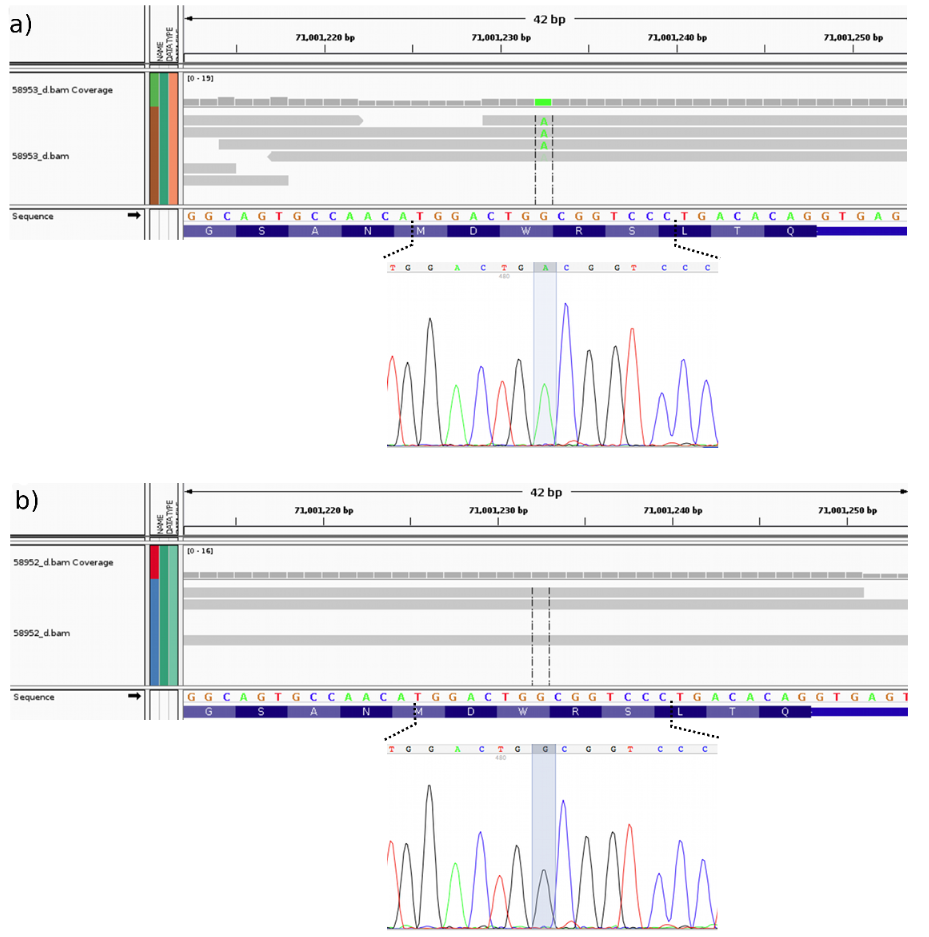

Supplement: Supplementary file 4 — Additional file 4: Visualisation of the p.W215X mutation in two apparently homozygous animals using the integrative genomics viewer (IGV, http://www.broadinstitute.org/igv/ ). Analysis of whole-genome re-sequencing data revealed a nonsense mutation in PLD4 as most likely causal for the disease of the affected calves. Re-sequencing confirmed that the mutation is homozygous in the affected calf. (PNG 258 KB) [file 12864_2014_6308_MOESM4_ESM.png]
