# Supplementary material for: A nonsense mutation in PLD4 is associated with a zinc deficiency-like syndrome in Fleckvieh cattle
Source: BMC Genomics. 2014 Jul 22;15(1):623. doi: 10.1186/1471-2164-15-623 (PMC4117962; doi:10.1186/1471-2164-15-623)
Supplement: Supplementary file 3 — Additional file 3: Genomic structure of the re-annotated PLD4 and INF2 genes. The genomic structure was predicted based on the University of Maryland UMD3.1 assembly of the bovine genome sequences [10] and the Dana-Farber Cancer Institute bovine gene index release 12.0 [33] by using GENOMETHREADER software tool. The GENOMETHREADER output was viewed and edited using Apollo sequence annotation editor [35]. (PDF 46 KB) [file 12864_2014_6308_MOESM3_ESM.pdf]

Genomic structure of the re-annotated *PLD4* and *INF2* genes

PLD4

No.	*length of exon (bp)*	*position chromosome 21*	*No.*	*length of intron (bp)*	*position chromosome 21*
1	50	70,995,733 – 70,995,782	1	1445	70,995,783 – 70,997,227
2	193	70,997,228 – 70,997,420	2	2190	70,997,421 – 70,999,610
3	200	70,999,611 – 70,999,810	3	619	70,999,811 – 71,000,428
4	184	71,000,429 – 71,000,613	4	183	71,000,614 – 71,000,796
5	121	71,000,797 – 71,000,917	5	202	71,000,918 – 71,001,119
6	128	71,001,120 – 71,001,247	6	824	71,001,248 – 71,002,071
7	201	71,002,072 – 71,002,272	7	639	71,002,273 – 71,002,911
8	140	71,002,912 – 71,003,051	8	110	71,003,052 – N's
9	166	N's – 71,003,789	9	74	71,003,790 – 71,003,863
10	97	71,003,864 – 71,003,960	10	312	71,003,961 – 71,004,272
11	470	71,004,273 – 71,004,742	11		

INF2

No.	*length of exon (bp)*	*position chromosome 21*	*No.*	*length of intron (bp)*	*position chromosome 21*
1	391	70,827,525 – 70,827,915	1	1,160	70,827,916 – 70,829,075
2	116	70,829,076 – 70,829,191	2	75	70,829,192 – 70,829,266
3	160	70,829,267 – 70,829,426	3	446	70,829,427 – 70,829,872
4	34	70,829,873 – 70,829,906	4	778	70,829,907 – 70,830,684
5	142	70,830,685 – 70,830,826	5	793	70,830,827 – 70,831,619
6	142	70,831,620 – 70,831,761	6	162	70,831,762 – 70,831,923
7	272	70,831,924 – 70,832,195	7	607	70,832,196 – 70,832,802
8	99	70,832,803 – 70,832,901	8	175	70,832,902 – 70,833,076
9	20	70,833,077 – 70,833,096	9	222	70,833,097 – 70,833,318
10	336	70,833,319 – 70,833,654	10	94	70,833,655 – 70,833,748
11	301	70,833,749 – 70,834,049	11	182	70,834,050 – 70,834,231
12	169	70,834,232 – 70,834,400	12	943	70,834,401 – 70,835,343
13	71	70,835,344 – 70,835,414	13	96	70,835,415 – 70,835,510
14	108	70,835,511 – 70,835,618	14	323	70,835,619 – 70,835,941
15	71	70,835,942 – 70,836,012	15	655	70,836,013 – 70,836,667
16	121	70,836,668 – 70,836,788	16	502	70,836,789 – 70,837,290
17	165	70,837,291 – 70,837,455	17	107	70,837,456 – 70,837,562
18	103	70,837,563 – 70,837,665	18	123	70,837,666 – 70,837,788
19	162	70,837,789 – 70,837,950	19	649	70,837,951 – 70,838,599
20	636	70,838,600 – 70,839,235	20	438	70,839,236 – 70,839,673
21	59	70,839,674 – 70,839,732			
